# Clinical Impact of an AI Decision Support System for Detection of Intracranial Hemorrhage in CT Scans

**DOI:** 10.1089/neur.2024.0017

**Published:** 2024-10-14

**Authors:** David Bark, Julia Basu, Dimitrios Toumpanakis, Johan Burwick Nyberg, Tomas Bjerner, Elham Rostami, David Fällmar

**Affiliations:** ^1^ Department of Neurosciences, Neurosurgery, Uppsala University Hospital, Uppsala, Sweden.; ^2^ Department of Surgical Sciences, Neuroradiology, Uppsala University, Uppsala, Sweden.; ^3^ Department of Radiology in Linköping, Linköping University, Linköping, Sweden.; ^4^ Department of Health, Medicine and Caring Sciences, Linköping University, Linköping, Sweden.; ^5^ Centre for Medical Image Science and Visualization (CMIV), Linköping University, Linköping, Sweden.; ^6^ Department of Neuroscience, Karolinska Institutet, Stockholm, Sweden.

**Keywords:** CNS, ICH, AI model, decision analysis, outcome analysis

## Abstract

This study aimed to evaluate the predictive value and clinical impact of a clinically implemented artificial neural network software model. The software detects intracranial hemorrhage (ICH) from head computed tomography (CT) scans and artificial intelligence (AI)-identified positive cases are then annotated in the work list for early radiologist evaluation. The index test was AI detection by the program Zebra Medical Vision—HealthICH+. Radiologist-confirmed ICH was the reference standard. The study compared whether time benefits from using the AI model led to faster escalation of patient care or surgery within the first 24 h. A total of 2,306 patients were evaluated by the software, and 288 AI-positive cases were included. The AI tool had a positive predictive value of 0.823. There was, however, no significant time reduction when comparing the patients who required escalation of care and those who did not. There was also no significant time reduction in those who required acute surgery compared with those who did not. Among the individual patients with reduced time delay, no cases with evident clinical benefit were identified. Although the clinically implemented AI-based decision support system showed adequate predictive value in identifying ICH, there was no significant clinical benefit for the patients in our setting. While AI-assisted detection of ICH shows great promise from a technical perspective, there remains a need to evaluate the clinical impact and perform external validation across different settings.

## Key Points

- The evaluated artificial intelligence software demonstrated a satisfactory positive predictive value in our external validation data set.
- The advantage in diagnostic time was minimal, with no significant differences between cohorts with and without hemorrhage across all prioritization categories.
- The software provided limited clinical impact, with no statistically significant advantage in time to escalation of the care level or in the time to acute surgery within 24 h.

## Introduction

In the acute care of patients with neurological conditions, computed tomography (CT) scanning of the head is a fundamental tool for rapid diagnostics and for selecting patients for neurosurgical consultation.^1^ In patients with intracranial hemorrhage (ICH), hematoma expansion and irreversible neurological damage can occur early and rapid diagnosis and surgical intervention can be life-saving.^2^ Therefore, a decision support system that automatically detects ICH on CT scans and highlights those cases in the radiologist’s worklist could save critical time.

Decision support for radiologists based on artificial intelligence (AI) has made immense advances and may soon become a cornerstone of clinical routine.^3^ Especially the detection of ICH on brain CT has very promising results.^4–6^ However, clinical implementation of these systems remains scarce, and there is need for substantial external validation in real-world settings in order to ensure sufficient performance in novel clinical environments.^7,8^ In fact, in a recent systematic review of external validation studies of deep learning algorithms for image-based radiological diagnosis,^9^ the vast majority of these algorithms demonstrated diminished diagnostic performance on external datasets, with some reporting a substantial performance decrease.

This study aimed to assess the clinical impact of a decision support system, Zebra Medical Vision—HealthICH+ version 3.1.24, for detecting ICH from both radiological and neurosurgical standpoints. This software uses three convolutional neural networks to analyze incoming CT scans for signs of hemorrhage, marking positive patients and alerting the radiologist. HealthICH was based on a 3D convolutional neural network trained to binary classify ICH on a dataset of around 100,000 scans. Every scan was labeled positive or negative for ICH, based on the accompanying radiology report. Earlier versions of the neural network had previously been trained in several steps on thousands of scans and validated.^10^ HealthICH was approved by the American Food and Drug Administration (FDA) in June 2019.^11^ As described in the FDA notification letter, the performance of the HealthICH device had also been validated in a stand-alone performance study, comprising ICH positive (*n* = 199) and negative cases (*n* = 228), as well as confounding imaging factors.^12^

The current evaluation took place at a tertiary care neuro-clinic in one of Sweden’s seven university hospitals (not involved in previous training or validation of the software). The primary endpoint was to assess the patient benefit with ICH generated by the supporting AI program. Secondary endpoints were the time benefit itself and the positive predictive value (PPV) of the program. An additional secondary endpoint was to evaluate whether false positive AI classifications contributed to false positive clinical assessments (i.e., misleading the primary radiological evaluation).

## Methods and Material

### Ethics approval

The study had approval from the Swedish Ethical Review Authority. Due to the acute nature of the examinations, the need for written patient consent was waivered by the Swedish Ethical Review Authority.

### Data collection

All head CT scans, except for stroke alarms, during two time periods of a total of five months (between December 2019 and April 2020) were included and evaluated. The gap in inclusion time was caused by maintenance on the AI server, without affecting the daily patient flow. Each patient was only included once (first instance). Of the 2306 included cases, 288 individual patients were positively annotated by the program (AI-positive), which were automatically flagged and noticeable in the radiologist’s worklist.

### Time benefit analysis

The referrals to head CT scans were categorized as “routine,” “subacute,” or “acute”, and the time point for scanning was defined as either “office hours” or “on call.” Time data was collected regarding scan completion and the first radiologists’ report. Time duration from AI-positive and AI-negative scans were compared.

### Radiological analysis

Positive AI annotations were compared to the contents of each final report, written by a specialist in neuroradiology. AI detections were classed as true positive and false positive, depending on the description in the report. Scans that were AI negative (*n* = 2018) were not re-evaluated in this retrospective study.

True positive findings were categorized into parenchymal, subdural, epidural, and subarachnoid hemorrhages. False positive findings were categorized into tumors, infarcts, artifacts, calcification, and unknown reason. PPV was calculated for intracranial bleedings *per se* and for “relevant diagnostic findings,” by combining true positives with cases with intracranial tumors without bleeding. Discrepancies between the preliminary report and final report were checked for false positive preliminary reports.

### Clinical benefit analysis

AI-positive patients who received reports faster than the median time for negative patients from the same referral category (“acute,” “subacute,” or “routine”) were identified as having “time benefit from AI detection,” suggesting potential subsequent clinical benefit. A neurosurgery resident systematically reviewed the medical records of all AI-positive cases that had a time delay below the median value for their respective category (*n* = 140) using an *a priori* designed data extraction form (Supplementary Data S1). The review assessed whether the diagnosis was known beforehand if the on-call neurosurgeon was summoned post-examination, and if the examination resulted in escalated care or emergency surgery within 24 h. The time gap between the examination and surgery was recorded.

### Statistical analysis

Statistical parameters were produced using R version 4.2.2 and graphs were produced using the ggplot2 library.^13^ Data skewness was determined by histogram as well as with Shapiro–Wilk test analysis. Because of data skewness, a significant difference in time benefit between different patient groups was analyzed with a two-sided Wilcoxon ranked sum test. Correlation between continuous variables was determined with linear regression analysis. The boxplot graph (Fig. 2) uses a square root *y*-scale to include extreme outliers.

## Results

**FIG. 1.**
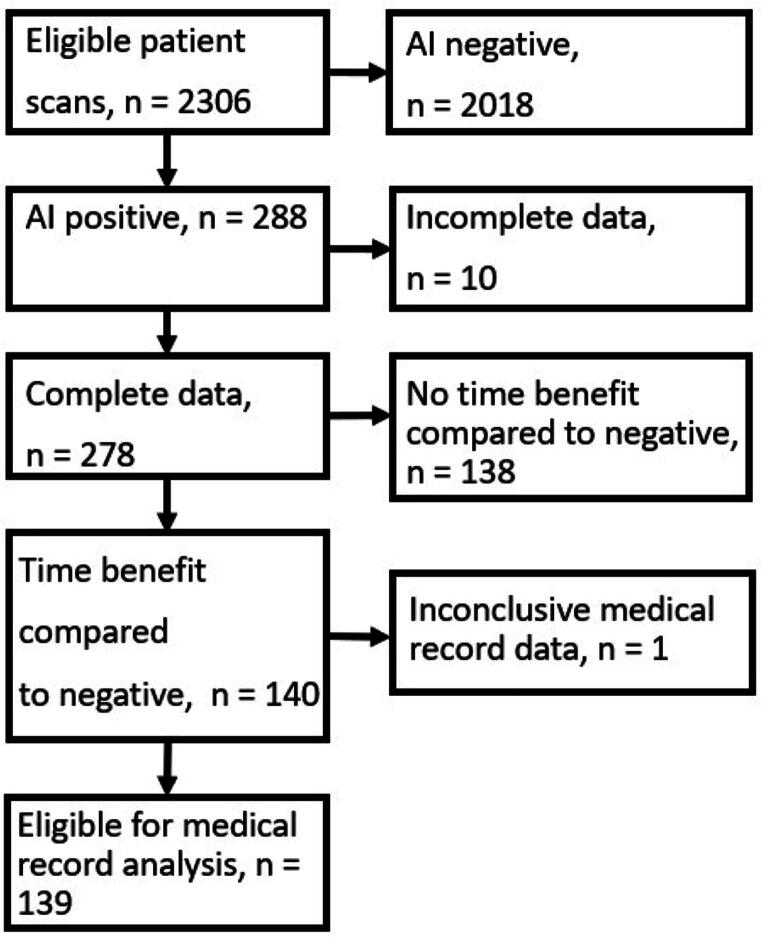
Flowchart of patient inclusion.

A summary of the exclusion criteria is displayed as a flowchart in Figure 1. After the exclusion of 10 cases with incomplete data, 278 AI-positive cases were evaluated by comparing the AI classification to the final report.

All cases with unclear or ambiguous findings (*n* = 45) were re-evaluated by a specialist in neuroradiology. Among the AI-positive cases, 10 (3.6%) had epidural hematomas, 37 (13.3%) had subarachnoid hemorrhages, 117 (42.1%) had subdural, and 65 (23.4%) had intraparenchymal blood. When several types of bleeding were present, the most dominant finding decided the classification. In the remaining 49 (17.6%) AI-positive cases no ICH was found, resulting in a PPV of 0.823 for detection of ICH. The false positive cases all had some other minor findings. Among the 49 false positive cases, many had other pathological findings that presumably triggered the artificially intelligent classification. Of these, 16 had intracranial tumors (without overt bleeding), four had infarcts and five were visually assessed as completely normal. The remaining 20 cases had some component (post-operative remnants, calcifications, artifacts, or abscess) that were assumed to be the source of the false positive classification. If the tumor was considered clinically relevant and thus a true positive finding for AI classification, PPV rose to 0.881.

In three of the AI-positive cases (1.1%), the preliminary report described an intracranial bleeding that was later negated in the final report. These were re-examined by a specialist in neuroradiology to evaluate if the primary radiologist was misled by the positive AI classification. All three cases had findings where “possible hemorrhagic components” were considered a plausible interpretation. Therefore, none of these false positive AI annotations were suspected to have misled the primary radiologist.

Median times in minutes between the time of the CT scan to the report (“time delay”) are listed in Table 1. None of the categories had a statistically significant difference between AI-positive and AI-negative scans.

**Table 1.** Time Benefits from AI-Detection in the Different Computed Tomography Priority Categories

Time of day	On call			Office hours		
Priority category	Acute	Subacute	Routine	Acute	Subacute	Routine
Median time	27.5/28.35	37.3/34.1	1421.9/1500.1	29.4/26.2	36.5/30.9	95.4/213.3
*n*	112/997	58/215	9/199	20/204	58/139	17/278
*p*	0.834	0.793	0.861	0.692	0.232	0.160

Median time in minutes for AI-positive/negative cases, on the group level for each of the six categories.

Total *n* = 2306.

AI, artificial intelligence.

The time between the CT scan to the report was also examined at the individual level for each AI-positive case compared with the median time of negative scans in the corresponding category (Table 2).

**Table 2.** Display of Time Benefit Windows in Different Priority Categories

Priority category:	Total	Acute	Subacute	Routine
Timebenefit >30 min:	17	0	0	17
Timebenefit 5–29 min:	99	49	49	1
Timebenefit <5 min:	24	21	3	0
Sum of category:	140	70	52	18

The number of patients with time benefit from AI-detection compared to the median within each of the three different priority categories. Time benefit in minutes is stratified into three arbitrary categories for presentational purposes.

AI, artificial intelligence.

AI-positive cases that had a time delay below the median value for their respective category (*n* = 140) were considered to have a time benefit and were eligible for further analysis. After excluding one patient with inconclusive records, 139 patients were screened (Fig. 1). The time benefit in these cases had a range of 0.05 to 624 min (median = 13.37, IQR = 12.91). Out of these 139 cases, 67 (48%) had a previously known diagnosis. In 27 patients (19%), the examination led to the on-call neurosurgeon being summoned, 17 (12%) to escalation of the level of care, and 8 (6%) to emergency surgery (within 24 h). Only 17 of these 139 patients had time benefit from AI detection of 30 min or more, and all these 17 were routine scans.

Of the 17 patients with ≥30 min time benefit, none resulted in summoning the on-call neurosurgeon, none led to escalation of the level of care, and none led to emergency surgery. Out of these 17 patients, 10 were control scans of known diagnoses and seven were newly diagnosed pathologies. Out of those seven cases, one was a newly diagnosed tumor, two were of small subdural hematomas, one was a post-surgical control, and three were false positives.

**FIG. 2.**
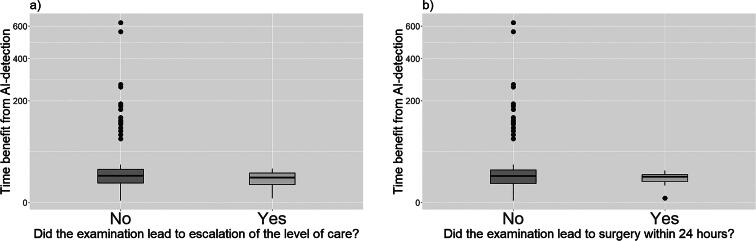
Time benefits from artificial intelligence (AI)-detection compared to clinical management of the patients. Note that the y-axis is square root transformed. **(a)** Comparison between groups with and without escalation of the level of care. There was no significant time benefit between the group that had escalation of the level of care compared to the group who did not, *p* = 0.1886. **(b)** Comparison between groups that did or did not go through emergency surgery. There was no significant time benefit between the group that had emergency surgery compared to the group who did not, *p* = 0.4611.

There was no significant time benefit from AI detection between the group that had emergency surgery compared to those who had not, *p* = 0.4611*,* or between the groups that had or did not have escalation of the level of care *p* = 0.1886 (Fig. 2). Among the eight patients who underwent surgery, there was one case where the time to surgery could not be extracted from the available medical records. In the remaining seven patients, there was no significant correlation between time benefit from AI detection compared with time to surgery (*p* = 0.498, *r*^2^ = 0.09619).

## Discussion

The first main finding was that, in an authentic clinical setting, the PPV in AI-annotated scans was 0.881 or 0.823 with or without considering tumors as relevant findings. This suggests that clinical implementation of AI detection is feasible and that further evaluation and clinical implementation of this software could be worthwhile. Furthermore, the field is in rapid development, and recent studies on two of the most popular software for automated detection of ICH, e-ASPECTS by Brainomix Limited and Rapid ICH by iSchemaView, Inc., have a PPV of 0.89 and 0.995 respectively.^14,15^

The second main finding was that among patients with time benefits from AI detection, the majority had a time benefit of less than 15 min compared with AI-negative patients from the same prioritization category. Thus, the observed clinical benefit from using AI for ICH detection may be limited. At the group level, there was no statistically significant time benefit between any category.

The third main finding was that there was no significant time benefit in patients who required escalation of care or acute surgery compared to those who did not. There was no significant correlation between time to surgery and time benefit from AI detection. Among the limited number of patients (*n* = 17) with more than 30 min of time benefit, there were no signs of clinical benefit, defined as elevated level of care or emergency surgery.

In our clinical setting, there was no measurable outcome benefit from triage assistance by the AI support system. An explanation for this could be that the referring physicians already prioritized the scans, with “acute” scans having a shorter median wait time than “subacute” and “routine” scans. Furthermore, in our setting, AI detection had limited impact on “routine” scans due to local conventions, such as CT technicians alerting radiologists about suspicious findings. However, in another clinical setting, it is possible that AI-positive “routine” scans could benefit more from AI prioritization for human evaluation. If a patient presents with a decreased level of consciousness, they’re typically prioritized for an acute CT scan, surgery, and escalated care. Those with better clinical statuses are often referred for routine scans, often revealing minor hemorrhages that may not necessitate escalated care or surgery. Acute patients’ scans are immediately evaluated by the attending neurosurgeon who decides on treatment, often bypassing the radiologist. In addition, software detection of larger, and thus symptomatic, hematomas is more likely compared to smaller ones and the clinical benefit would thus be largest in hematomas that the software is likely to find, while small hematomas that the software might miss is less likely to have a large clinical impact. The time advantage from AI detection was higher in patients with a longer time to acute surgery, especially in nonacute cases discovered in nonacute scans. During office hours, median times for acute and subacute cases were slightly *longer* for AI-positive cases (Table 1). This is counterintuitive but probably reflects, to some extent, patients that are well known for the staff and the radiologist, and where the report is not rushed due to the AI detection.

There was no certain case in this study where a false positive classification by the AI misled the interpreting radiologist to a false positive report. When the program was implemented, the possibility of false positives was discussed which probably was an important contextual factor. Regarding the radiologist’s confidence in working in conjunction with AI, the availability of reliable saliency maps would probably be of high importance.^16^ To provide additional context, Kundisch et al.^17^ examined a similar software model in a German cohort and found discrepant results between AI and a radiologist to be only 162 out of 4946 (3%) with some misclassification by both the AI and the primary radiologist.

In a recent systematic review and meta-analysis, Jørgensen et al.^3^ conclude that detection of ICH remains a promising prospect of conventional neural networks and that sensitivity and specificity often is on par with radiologists. Furthermore, the implementation of AI-based solutions for the detection of ICH could improve the workflow and workload of radiologists and hopefully reduce the time to diagnosis and improve clinical outcomes. However, they conclude that there is a need for additional studies, especially including external validation. Clinical implementation of AI tools in a variety of fields remains to be realized. In fact, in a systematic review by Lam et al.,^18^ the authors conclude that there is a very limited number of randomized clinical trials for the implementation of clinical AI tools and that while a majority of these reports improved primary endpoints compared to standard care, very few show clinically relevant improvement.

## Limitations

The main limitation of this study was its single-center, open-label nature and the fact that negative cases could not be included. However, the main focus of this study was the clinical management of true positive hemorrhages and false negative findings would likely bring low clinical yield. The limited number of patients requiring acute surgery prevents definite conclusions about the correlation between AI-assisted prioritization and time to surgery. Another limitation was the retrospective definition of time benefit from AI-detection, which could potentially overlook individual case details. Future studies should ideally randomize groups for AI evaluation and compare them within each priority category.

The effects and potential benefits of an intervention in a real-world clinical setting are heavily dependent on the specific workflow as well as the composition of the patient population. The same AI-based triage software might have more impact in a smaller hospital where the CTs are frequently initially read by inexperienced readers or non-radiologists. When assessing an AI-based software, the potential benefit will be different in various settings and results are not universally generalizable. The present study represents the reality of a medium-sized tertiary university hospital with a complete neurosurgical organization.

Furthermore, the potential benefits of using an AI algorithm in the clinical workflow must be weighed against costs and risks. In addition to the cost of the algorithm, the cost of implementation needs to be considered. In view of the recent developments, it is fair to assume that in the (near?) future these kinds of algorithms are likely to be a large part of the workflow in a radiology department. It will therefore be important to choose algorithms that solve real problems in the organization and mitigate the costs and risks. It will likely be necessary to have a structure for the use and implementation of algorithms in a department by, for example, using portals that give access to multiple algorithms that can be implemented in similar ways and that algorithms interact with users in the clinical workflow in comparable and efficient ways.

## Conclusions

The main conclusion from this study is that although a number of patients with AI-positive findings had a shortened time delay between CT scan and report, this time benefit did not result in any measurable clinical benefit for the patients. Although future use of machine learning tools in health care is promising, its implementation must be intentionally adapted to meet real-life needs in the clinical setting, and potential benefits should be systematically evaluated.

## Abbreviations Used

AI: Artificial intelligence
CT: Computed tomography
ICH: Intracranial hemorrhage
IQR: Interquartile range
PPV: Positive predictive value
