# Supplementary material for: Clinical Impact of an AI Decision Support System for Detection of Intracranial Hemorrhage in CT Scans
Source: Neurotrauma Rep. 2024 Oct 14;5(1):1009–15. doi: 10.1089/neur.2024.0017 (PMC11491571; doi:10.1089/neur.2024.0017)
Supplement: Supplementary Data S1 [file neur.2024.0017_supplementary_data.pdf]

AI decision support system Zebra data collection form

1. Anonymized ID:

2. Main questions:

	Yes	No	Unknown
Previously known diagnosis before the examination?			
Was the on-call neurosurgeon summoned after the examination?			
Was there escalation of the level of care after the examination?			
Was there acute surgery (within 24 hours) after the examination?			

3. If the patient underwent surgery, how many hours between the examination and the surgery?

4. Optional comment:

5. Potential impact on patient outcome:
